# Glycosylation of HDL-Associated Proteins and Its Implications in Cardiovascular Disease Diagnosis, Metabolism and Function

**DOI:** 10.3389/fcvm.2022.928566

**Published:** 2022-05-27

**Authors:** Eduardo Z. Romo, Angela M. Zivkovic

**Affiliations:** Department of Nutrition, University of California, Davis, Davis, CA, United States

**Keywords:** glycosylation, high-density lipoprotein (HDL), O-glycosylation, N-glycosylation, ApoA-I, APOC3, APOE

## Abstract

High-density lipoprotein (HDL) particles, long known for their critical role in the prevention of cardiovascular disease (CVD), were recently identified to carry a wide array of glycosylated proteins, and the importance of this glycosylation in the structure, function and metabolism of HDL are starting to emerge. Early studies have demonstrated differential glycosylation of HDL-associated proteins in various pathological states, which may be key to understanding their etiological role in these diseases and may be important for diagnostic development. Given the vast array and specificity of glycosylation pathways, the study of HDL-associated glycosylation has the potential to uncover novel mechanisms and biomarkers of CVD. To date, no large studies examining the relationships between HDL glycosylation profiles and cardiovascular outcomes have been performed. However, small pilot studies provide promising preliminary evidence that such a relationship may exist. In this review article we discuss the current state of the evidence on the glycosylation of HDL-associated proteins, the potential for HDL glycosylation profiling in CVD diagnostics, how glycosylation affects HDL function, and the potential for modifying the glycosylation of HDL-associated proteins to confer therapeutic value.

## Introduction

It has been established across multiple cohorts that high density lipoproteins (HDL) are atheroprotective (1–4). HDL are complex, heterogeneous nanoparticles, with various subclasses, comprised of numerous functional proteins and lipids (5), and have more recently been shown to be highly glycosylated (6) and structurally and compositionally variable in various physiological and pathological states (7, 8). Owing to this high heterogeneity, HDL particles have diverse biological functions including immunomodulatory, anti-inflammatory, antioxidant, antithrombotic, and anti-proteolytic functions among others, which are dependent on their composition (9–13). Protein and lipid composition, as well as particle structure and size, are important known factors driving differences in HDL functional capacity. The role of glycosylation in the differential functionality of HDL particles has only recently started to emerge.

**Figure 1:**
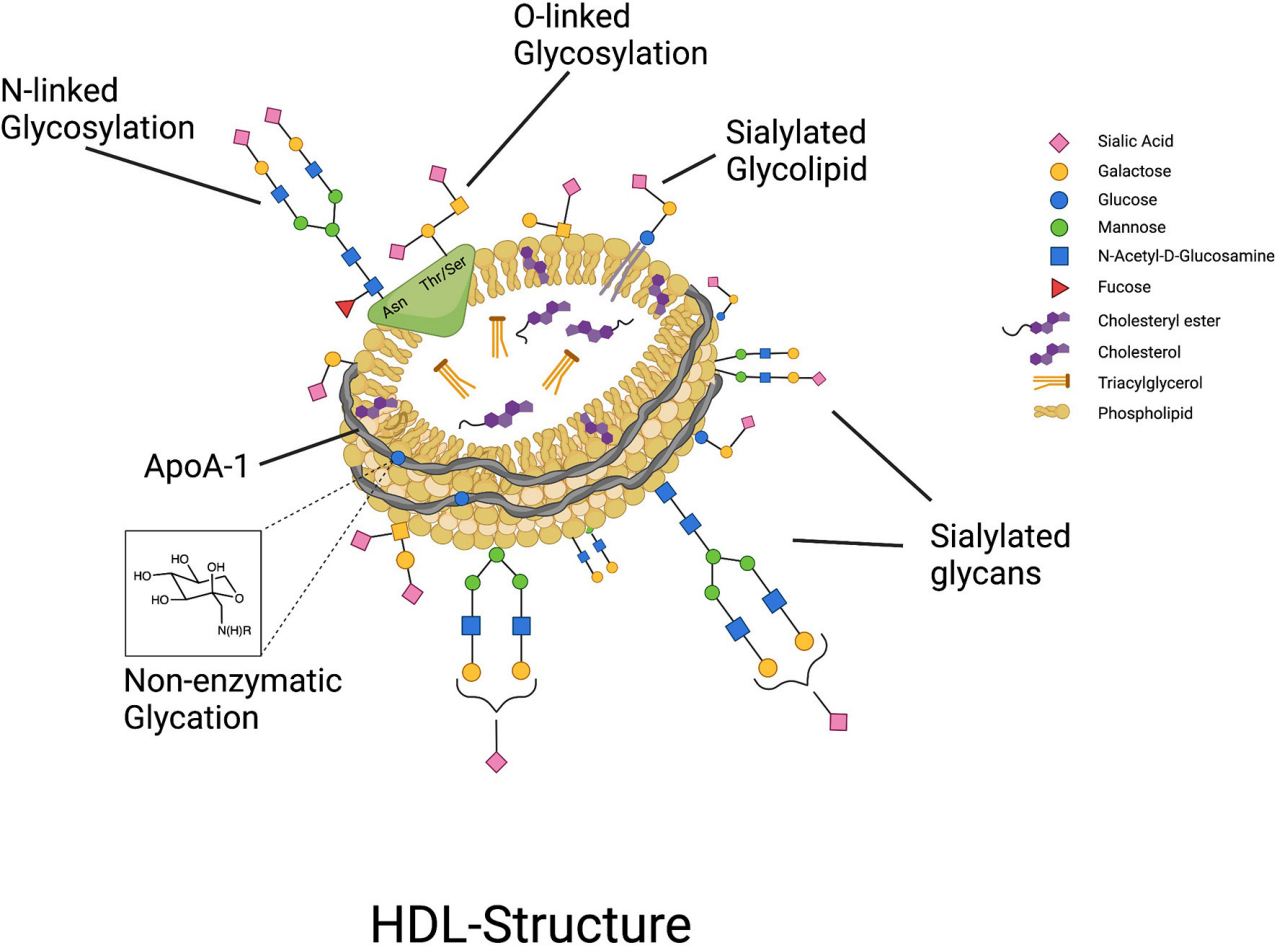
HDL particles are highly glycosylated, containing both glycoproteins that can be N- and O-glycosylated, and glycolipids, with glycan groups added enzymatically through highly regulated cellular processes. In contrast, non-enzymatic glycation of proteins such as ApoA-I can occur under conditions of hyperglycemia.

Protein glycosylation is generally an enzymatically driven post-translational modification of newly biosynthesized proteins that occurs in the endoplasmic reticulum and Golgi apparatus where sugars are attached to proteins by N- or O-linkages, forming glycans (14). N-glycans are attached to a nitrogen atom on the asparagine moiety of the protein whereas O-glycans are bound to the oxygen atom of either threonine or serine (15). Glycans contribute to various biological capacities including protein folding, receptor binding, enzyme activity, and physical properties by lending charge to the protein, and are vastly particular to the type, extent, and specific site of glycosylation (15–19). Protein glycosylation functions as a biological language and is important for biological particle self- and non- self-recognition, molecule transport, and endocytosis (20). In the last 8 years since it was first demonstrated that HDL are highly glycosylated, and specifically sialylated particles (6) (Figure 1), there has been a steady increase in the evidence pointing to an important connection between the glycosylation of HDL-associated proteins, and the overall functionality of HDL particles. In this review paper we will discuss the current state of the evidence on the glycosylation of HDL-associated proteins, specifically, where we stand in terms of development of cardiovascular disease (CVD) diagnostics using HDL-glycosylation profiling, how glycosylation of HDL proteins affects HDL function, and the potential for modifying the glycosylation of HDL-associated proteins to confer therapeutic value.

## HDL Glycosylation Profiling for Diagnostic Purposes

One of the problems with HDL particle analysis for diagnostic purposes has been the extreme complexity of these particles and the lack of resolution of older measurement tools. For example, although high HDL-cholesterol (HDL-C) concentrations have been found to be protective against CVD, several large recent studies demonstrated that the relationship between HDL-C concentration and adverse health outcomes tends to follow a U-shaped curve, with both low HDL-C and very high HDL-C being associated with increased cardiovascular (CV) events (21–23). Clearly, it is not simply the measurement of the total amount of cholesterol carried within HDL that is diagnostic, but rather some other aspect of HDL that is critical, whether it be compositional, structural, or functional.

For more sophisticated measurements of HDL structure, composition, and function, it is imperative to first isolate the HDL particles and purify them from other potentially contaminating components. Because HDL particles are so small (7–12 nm in diameter) as to overlap with many plasma proteins in terms of their size (e.g., ferritin), and because they are close in density to other lipoprotein particles and even extracellular vesicles, they are difficult to isolate and purify. According to multiple proteomic studies HDL could carry as few as 12 key proteins or up to an excess of 200 proteins (24, 25) depending on how they are isolated (24, 26, 27). Various methods, and combinations of these methods, have been used to isolate HDL including ultracentrifugation, size exclusion chromatography, immunoaffinity precipitation, and asymmetrical flow field flow fractionation. More recently, methods combining these different approaches have been used to improve the overall yield and purity of HDL particles while preserving their structural and functional integrity (28–30), including an optimized, validated method using sequential flotation density ultracentrifugation followed by size exclusion chromatography which yields highly purified HDL fractions (5).

Once HDL particles are isolated, the analysis of their glycosylation status can be performed. Pioneering work in lipoprotein glycobiology establishing analytical methods for profiling the glycome of HDL particles revealed for the first time that HDL has both N- and O-linked glycosylation and is distinctly highly sialylated (6). Glycosylation analysis is a complex chemical approach traditionally using mass spectrometry combined with various extraction methods such as enzymatic digestion, chemical cleavage, and liquid chromatography (31–35). HDL glycosylation can be profiled in several ways: (1) the glycans can be enzymatically or chemically cleaved, followed by mass spectrometry (6), (2) site-specific glycoprofiling can be performed by tandem mass spectrometry analysis of protease-digested samples (6, 36, 37), and (3) hydrophilic interaction liquid chromatography profiling can be performed, which uses a combination of the three main types of liquid chromatography for separation and profiling of glycan-containing peaks (38, 39).

To date, no large studies examining the relationships between HDL glycosylation profiles and CVD outcomes have been performed. However, small pilot studies provide promising preliminary evidence that such a relationship may exist. For example, in a small pilot study performed by our group, differences in HDL glycan composition were able to differentiate between individuals at equal risk for CVD based on clinical parameters (i.e., total cholesterol, low-density lipoprotein-cholesterol (LDL-C), HDL-C, etc.) who were found to have arterial occlusion vs. not (37). The role of HDL glycosylation in CV health is starting to be recognized as a promising new research field (40). Larger cohort studies investigating the relationships between HDL glycoprofiles and CV outcomes across factors including age, sex, and ethnicity are needed, and have the potential to add greatly to our ability to detect individuals at risk for CVD earlier when disease prevention measures are the most likely to be effective.

## How Glycosylation of HDL Proteins Affects HDL Function

Most of the known HDL-associated proteins are glycosylated, and only a few are found to be non-glycosylated. In Table 1 we provide information on the N- and/or O-glycosylation status, sites of attachment, and number of unique glycans attached for several HDL proteins for which this information has been confirmed by extensive MS/MS analysis. Whereas, here are many putative sites for O-glycosylation (i.e., any Ser or Thr residue) on any given protein, whether O-glycans are actually attached must be confirmed by advanced MS analysis. Thus, although several HDL-associated proteins, such as ApoC-I have Ser or Thr residues that could in theory be O-glycosylated, in Table 1 we report only those that have been demonstrated to beO-glycosylated by MS measurement of isolated HDL fractions derived from a starting volume of 500 uL of plasma. It is possible that some proteins (e.g., PLTP) are present at such low abundance in isolated HDL that their glycoforms fall below the limits of detection. Thus, to further investigate the glycosylation status of these low-abundance HDL proteins future experiments involving enrichment for these proteins will be required. Other proteins, such as ApoA-I, have been reported to be glycosylated in the literature, however, we do not include it in Table 1 because based on detailed MS analysis the O-glycosylation could not be confirmed. In the following section we review what is currently known about the impact of glycosylation of several key HDL-associated on overall HDL metabolism and function, for which there is currently information. As the field evolves this list will doubtless grow and a more comprehensive picture of the extent and diversity of glycans attached to HDL-associated proteins will emerge.

**Table 1:** Glycosylation status of HDL-associated proteins with confirmed glycosylation sites.

**Protein**	**N-glycans**	**O-glycans**	**Sites of attachment**
Alpha-1-antichymotrypsin (AACT)	8	0	Asn106, Asn127, Asn271
Alpha-1-antitrypsin (A1AT)	18	0	Asn70, Asn107, Asn271
Alpha-1B-glycoprotein (A1BG)	1	0	Asn179
Alpha-2-HS-glycoprotein (FETUA or A2HSG)	17	2	Asn156, Asn176, Thr346
Apolipoprotein A-II (APOA2)	0	4	Ser35, Ser88, Thr95
Apolipoprotein C-III (APOC3)	0	21	Thr94
Apolipoprotein D (APOD)	28	0	Asn65, Asn98
Apolipoprotein E (APOE)	0	40	Ser215, Thr307/Ser308*, Ser76/Thr83*, Ser129/Thr130*, Thr194, Ser197, Ser263, Thr289/Ser290* Ser296 (25, 41)
Apolipoprotein F (APOF)	0	3	Ser269, Thr273/Thr27*
Apolipoprotein M (APOM)	9	0	Asn135
Clusterin (CLUS or APOJ)	10	0	Asn86, Asn291, Asn374
Complement C1s subcomponent (C1S)	2	0	Asn174
Complement C3 (C3)	4	0	Asn85
Hemopexin (HPX)	6	0	Asn187, Asn453, Asn240/Asn246*
Heparin cofactor 2 (HCF2)	2	0	Asn49
Kininogen-1 (KNG1)	4	0	Asn169, Asn205
Lecithin-cholesterol acyltransferase (LCAT)	1	0	Asn108
Serum amyloid A-4 (SAA4)	7	0	Asn94
Serum paraoxonase/arylesterase 1 (PON1)	8	0	Asn253, Asn324

*Proteins included in this table include only those with glycosylation sites confirmed to actually express glycans at those sites by mass spectrometry analysis of isolated HDL fractions from a starting volume of 500 uL of plasma, as described in (25). HDL-associated proteins that have been reported to be glycosylated previously, and/or have putative sites but that either could not be confirmed by mass spectrometry or are present at low abundance such that they fall under the limit of detection, are not reported in this table*.

^*^For these sites, the site of attachment could not be disambiguated thus both possible attachment sites are reported.

### Apolipoprotein A-I

ApoA-I, the major structural, defining HDL apoprotein accounting for around 70% of total HDL protein mass, plays a key role in lipid and cholesterol metabolism and is highly associated with cardioprotection (42). Despite ApoA-I being reported to possibly be glycosylated (43–45) extensive mass spectrometry-based (MS)-based profiling demonstrated that there is no evidence of ApoA-I glycosylation (25). ApoA-I does not have the consensus sequence for N-glycosylation (AsnXxxSer/Thr/Cys, where Xxx can be any amino acid except proline), and whereas O-glycosylation is possible on any serine or threonine residue, detailed analysis of isolated HDL has not yielded any confirmed O-glycosylated peptides on ApoA-I. It is possible that ApoA-I O-glycosylation can occur in certain conditions or disease states, however MS-based analysis has never confirmed the existence of this to date. ApoA-I can, however, be non-enzymatically or chemically glycated (44), which has been found to be deleterious for its function.

### Apolipoprotein A-II

ApoA-II is the second most abundant HDL apoprotein, representing as much as 20% of total protein mass (42), and has been shown to have important implications for CV health though results were historically inconsistent and controversial. For instance one early study showed that low serum ApoA-II was a marker of atheroprotection in patients with non-insulin-dependent-diabetes mellitus (46) but conversely another study showed that elevated levels of ApoA-II were proatherogenic (47). However, more recently a large prospective study (*n* = 912) showed that ApoA-II was indeed inversely associated with future risk for coronary artery disease (CAD) and was exerting antiatherogenic properties (48). ApoA-II binds to phospholipid transfer protein (PLTP) on HDL (49), suggesting that it plays an important role in the remodeling of HDL particles. ApoA-II contributes to structural properties of HDL (50) and its presence on HDL enhances ATP-binding cassette transporter-1 (ABCA-1)-mediated efflux, suggesting that ApoA-II can contribute to structural changes in ApoA-I, and improve functionality of the HDL particle (51). Like ApoA-I, ApoA-II does not contain the consensus sequence for N-linked glycosylation, however it has been shown to be O-glycosylated (25, 52). The glycosylation of ApoA-II contributes to its association affinities since sialylated ApoA-II preferentially associates with smaller HDL whereas non-sialylated ApoA-II associates with all sizes of HDL (53). In a recent study in patients who were equally at risk for CAD based on traditional biomarkers and who were then diagnosed as either having CAD or not using diagnostic coronary arteriography, ApoA-II was significantly lower in CAD patients compared to patients without CAD (7). In children given a lipid rich dietary supplementation there was no difference in ApoA-II glycosylation between groups, but the analysis did confirm that ApoA-II indeed had multiple glycoforms (25). It is currently unknown what the role of glycosylation in ApoA-II function is, and whether the extent of sialylation drives the binding of ApoA-II to smaller HDL particles or whether higher sialylation is reflective of a particular pathway of metabolism that is linked with the production of small particles.

### Apolipoprotein C-III

ApoC-III is a critical metabolic protein whose glycosylation status has long been known to be an important determinant of its function. ApoC-III is a small (8 kDa) O-glycosylated apoprotein whose glycans can be capped with 0, 1, or 2 sialic acids and thus is often denoted as, ApoC-III_0_, ApoC-III_1_, and ApoC-III_2_ accordingly. Because of the negative charge conferred by the sialic acids the ApoC-III glycoforms have differential migration on gel (18), which enabled the study of its glycosylation much earlier than more advanced MS-based tools became available. ApoC-III is synthesized in the liver and intestine and found on very-low-density-lipoproteins (VLDL), chylomicrons, LDL and HDL and is a multifunctional protein whose primary functions are to hinder apolipoprotein E (ApoE) mediated hepatic uptake of lipoproteins, and to inhibit lipoprotein lipase, a key enzyme that catalyzes the hydrolysis of triacylglycerols from lipoproteins to free fatty acids and monoacylglycerol fragments (54). ApoC-III has gained considerable attention due to its relationship with CV health and the strong correlation with ApoC-III overexpression and CVD due to its involvement in hypertriglyceridemia (55, 56).

Though the association between elevated ApoC-III concentration and CVD has been established for some time, the focus has been primarily on the role of ApoC-III in VLDL metabolism, however, recently a relationship between ApoC-III and HDL has emerged. For example, CVD patients have increased HDL ApoC-III content (57, 58). Changes in sialyation in the more common glycoforms of ApoC-III have been observed in multiple conditions including uremia, obesity, kidney disease, cancers and diabetes (8, 59–62). The enzyme from the GalNAc-transferase family Golgi-localized polypeptide N-acetyl-D-galactosamine-transferase 2 isozyme (GALNT2) initiates the first step in the O-glycosylation of ApoC-III, as well as several other lipoprotein-associated targets including ApoE, PLTP, and angiopoietin-like 3 (ANGPTL3) (63). Loss of function of GALNT2 was found to be associated with extremely low HDL concentrations (64), highlighting the importance of O-glycosylation of critical apoproteins and related proteins involved in lipoprotein remodeling in HDL metabolism. Elevated circulating levels of triglycerides (TG) are a risk factor for CVD (65) which is positively correlated with circulating ApoC-III concentrations (66, 67). High-throughput mass spectrometric immunoassay found that increased plasma TG levels were associated with higher ratio of ApoC-III_1_ over ApoC-III_2_ (68). Importantly, it is already well-known that the sialylation state of ApoC-III associated with LDL particles is responsible for its binding affinity to cell surface receptors, with ApoC-III_2_ being preferentially cleared by heparan sulfate proteoglycans and conversely ApoC-III_1_ being more effectively cleared by the LDL receptor and other receptors in the LDL receptor family (69). It is currently unknown whether and how the sialylation state of ApoC-III associated with HDL particles influences the binding of those HDL to cell surface receptors.

The glycosylation of ApoC-III is more complex than was previously thought. In addition to the known glycosite at position Alanine-74 (Ala)-74 (70) and the three possible non-sialylated and sialylated glycans attached at this site (6), our group identified a total of 20 glycoforms most of which were fucosylated and nearly half were sialylated (15). Interestingly, 13 unique glycoforms of ApoC-III were significantly enriched in HDL particles compared to serum, with the HDL-associated glycoforms being more highly sialylated (15). These findings suggest that either ApoC-III glycosylation state modifies its affinity for a specific lipoprotein class, or that the metabolism of ApoC-III and its exchange between the circulating lipoproteins is reflected in its glycosylation. Research is needed to better understand the mechanisms driving these intriguing findings about the links between ApoC-III glycosylation and its association with HDL vs. the ApoB containing lipoproteins, and the unique role of ApoC-III in HDL particle metabolism.

In a recent study comparing the site-specific glycosylation of ApoC-III in patients across the spectrum from healthy, to those with metabolic syndrome to diabetic patients with chronic kidney disease on hemodialysis, ApoC-III was differentially glycosylated in patients with metabolic syndrome and diabetic hemodialysis compared to controls (37). Patients with chronic kidney disease who were on hemodialysis and patients with metabolic syndrome had HDL that were significantly more enriched in ApoC-III especially in di-sialylated ApoC-III (ApoC-III_2_) compared to the control group (37). Importantly, HDL ApoC-III glycosylation was able to distinguish between HDL that suppressed vs. increased IL-6 secretion by monocytes stimulated with lipopolysaccharide (LPS), when clinical biomarkers such as total cholesterol, LDL cholesterol, C-reactive protein (CRP), glucose and blood pressure were not discriminatory in this immunomodulatory ability (37). These intriguing preliminary findings suggest that ApoC-III glycosylation may play an important role in directing the immunomodulatory capacity of HDL particles.

### Apolipoprotein E

ApoE may well be one of the most influential proteins in lipoprotein biology, and in metabolic health overall. Genome-wide association studies across multiple geographic regions have irrefutably identified APOE, which directs lipoprotein metabolism both peripherally and in the central nervous system, as the single strongest genetic marker of extreme longevity across multiple, multi-ethnic cohorts (70). APOE genotype is a major risk factor for a number of age-related pathologies including CVD and Alzheimer's disease (71, 72). ApoE exists in three isoforms, ApoE2, ApoE3, and ApoE4, with ApoE4 conferring increased risk for both CVD and Alzheimer's (73–75). Importantly, it is well-known that compared to ApoE3 the ApoE4 isoform has a reduced ability to induce cholesterol efflux (76, 77), and has a higher binding affinity for VLDL than HDL particles, altering its metabolic fate (78). Unlike the intracellular fate of ApoB-100, which is largely degraded upon uptake via the LDL receptor, as much as 80% of ApoE internalized as part of VLDL particles is recycled and re-secreted as part of HDL particles (79). This recycling and re-secretion pathway is not exclusive to hepatocytes, and instead has been demonstrated to occur across a wide variety of cell types (79). Importantly, when internalized as part of TG-rich lipoproteins via receptors in the LDL receptor family, ApoE4 is more likely to be retained in the cell than recycled and re-secreted as part of HDL particles compared to ApoE3, resulting in diminished concentrations of ApoE4 in circulation and reduced cholesterol efflux (76). The endocytic vesicles involved in ApoE recycling were identified to contain sialyltransferase enzymes (80), suggesting that addition of sialic acid residues to ApoE glycan structures may be a critical step in directing ApoE from internalized TG-rich particles to re-secreted HDL particles. In support of this hypothesis, it has been found that HDL-associated ApoE is more highly sialylated than VLDL-associated ApoE (81).

ApoE was found to be glycosylated in 1979 (82), with 6 sialylated glycoforms identified (83). ApoE does not contain the consensus amino acid sequence for N-linked glycosylation, and instead is O-glycosylated with mucin-type glycans at the originally characterized site at Threonine^194^ (Thr194), which is not essential for ApoE secretion (84). More recently, additional glycosylation sites have been identified, including one at Thr^212^ (85), and 3 additional sites were identified at Serine^290^ (Ser^290^), Thr^289^ and Ser^296^ in ApoE secreted by macrophages isolated from peripheral blood mononuclear cells of a single donor with ApoE3/E3 genotype (86). It was recently shown that ApoE in fact has two more glycosites, for a total of 7 mucin-type O-glycosylation sites, with glycans ranging from simple GlcNAc to biantennary structures containing sialylation and fucosylation (87). Evidence regarding the importance of ApoE glycosylation in lipoprotein function is starting to emerge, building on the established evidence that ApoE structure impacts the metabolism of lipoproteins (41, 76). An aberrantly glycosylated variant of ApoE causes defective binding to the LDL receptor (88). ApoE is highly sialyated when associated with HDL compared to serum, and its sialylation state is involved in mediating ApoE's binding affinity to HDL vs. VLDL (81, 89). ApoE glycosylation was shown to be considerably different in cerebral spinal fluid (CSF) than in serum (90) and its extent of sialylation in CSF affects ApoE binding to amyloid beta, thus influencing the development of plaque formation and Alzheimer's disease (91) and suggesting that glycosylation of ApoE may be tissue-specific (90). Importantly, it was recently demonstrated that site-specific glycoprofiles of HDL-associated ApoE are correlated with HDL functional capacity (87), strongly suggesting that ApoE glycosylation is important for HDL function. ApoE isoform-specific glycoprofiling has not yet been performed and will likely be important in distinguishing ApoE genotype-specific effects on disease risk.

### Alpha-1 Antitrypsin

A1AT is an acute phase protein mainly synthesized by the liver, which acts as a protease inhibitor, and which has been shown to increase dramatically during inflammation and has also been found to persist post infection (92). Recent work showed that statins can also induce A1AT concentrations, and that association of A1AT with HDL protects the protein and enhances its anti-proteolytic activity in the context of the highly oxidative environment of the acute phase response (93). Post translational modifications of A1AT contribute to changes in conformation that may influence its function (94). Differential glycoforms of A1AT have been reported in patients with various types of lung cancers and are used in lung cancer diagnosis (95). Sialylation variations of A1AT have also been observed in patients with COVID-19 (96).

A1AT is N-glycosylated, and its site-specific glycosylation profiles differ when associated with HDL compared to serum (15). A1AT glycosylation is critical for its secretion by monocytes (97), is differential between serum and hepatocytes (98), and has increased fucosylated biantennary glycans in the serum of hepatocellular carcinoma patients (99). The site-specific glycosylation profiles of A1AT were highly differential between diabetic chronic kidney disease patients on hemodialysis compared to patients with metabolic syndrome and healthy controls: kidney disease patients had a higher proportion of monofucosylated to non-fucosylated glycans, and a lower proportion of di-sialylated glycans on A1AT (37). In the same study, HDL particles that attenuated the amount of Interlukin-6 (IL-6) secreted by LPS-stimulated monocytes had higher amounts of A1AT as well as lower amounts of several disialylated glycans across multiple sites, suggesting A1AT and its specific glycoprofile are involved in mediating HDL immunomodulatory function (37). A disialylated A1AT glycopeptide was also positively correlated with cholesterol efflux capacity in healthy young adults (87), and in young children from Ghana (25). These findings suggest an important connection between HDL A1AT glycosylation, particularly disialylated A1AT glycans, and HDL functionality.

### Alpha-2-HS-Glycoprotein

A2HSG is a hepatically derived protein found in plasma and associated with HDL particles (24). Several studies have shown that A2HSG is critically important for CV health (100–103), playing a particularly important role in preventing vascular calcification, and emerging as an independent risk factor of CVD and all-cause mortality (100). A2HSG is differentially glycosylated in patients with chronic pancreatitis and pancreatic cancer (104). Site-specific analysis of HDL-associated A2HSG revealed that it is highly sialylated and decorated with both N- and O-glycans at multiple sites (6). In patients with chronic kidney disease HDL were enriched with non-sialylated A2HSG, and non-sialylated A2HSG was enriched in HDL particles that enhanced IL-6 secretion by LPS-stimulated monocytes (37). Interestingly, A2HSG concentrations were lower in HDL compared to serum but specific glycoforms were significantly more enriched in HDL than in serum (15). Multiple A2HSG glycopeptides were positively correlated with HDL cholesterol efflux capacity and immunomodulatory capacity in healthy adults (87), and in young children in Ghana supplemented with a lipid-based nutrient supplement (25).

### Lecithin-Cholesterol Acyltransferase

LCAT functions as a key enzyme in reverse cholesterol transport and HDL particle maturation by esterifying free cholesterol with a fatty acid from phosphatidylcholine (lecithin), which allows HDL particles to carry a larger cholesterol load as cholesteryl esters (CE) in the core of the particle (105). LCAT is strongly linked with CV health and disease (106). ApoA-I is a potent activator of LCAT (107). Mutations in the LCAT gene lead to altered function of the enzyme resulting in elevated levels of TG and reduced HDL-C, which can lead to atherosclerotic pathology (108). The glycosylation of LCAT has been known since the 1990's, with both N-linked and O-linked glycoforms identified (109, 110), and with important implications for LCAT function (111). The glycosylation of LCAT is critical for its structural stability and function (112). Loss of glycosylation at several sites resulted in loss of function but loss of glycosylation at site 408 increased the activity of the enzyme (113). Desialylation of LCAT by neuraminidase resulted in considerable alteration of LCAT activity, reducing cholesterol esterification and concomitantly reducing the size of HDL (114). Depending on LCAT glycotype LCAT binds preferentially to HDL or ApoB-containing lipoproteins (115). These findings provide strong evidence that LCAT glycosylation is imperative for overall lipoprotein metabolism as well as cholesterol efflux and transport globally, as well as metabolism and efflux capacity of HDL particles in particular.

### Cholesterol Ester Transfer Protein

CETP is a critical mediator of lipid transfer between HDL and ApoB-containing lipoproteins, which in the context of high TG concentrations, transfers CE from HDL in exchange for TG from ApoB-lipoproteins, thereby enriching HDL particles with TG and altering their metabolism (116, 117). Loss of function genetic mutations in CETP and lower concentrations of CETP are associated with lower LDL-C and increased HDL-C, and lower risk of CVD, which has made CETP a major pharmacological target for CVD and atherosclerosis prevention (116, 118). CETP is highly sialylated with four N-linked glycoforms (119). A major form of serum CETP lacking glycosylation at Asparagine341 (Asn341) was shown to have markedly increased functionality compared to other forms (119, 120). Defective sialylation of CETP in heavy alcohol drinkers showed a significant reduction in the function of CETP compared to controls (121). Patients with a congenital disorder of glycosylation of the glycosyltransferase enzyme beta-1,4-galactosyltransferase 1 have defectively glycosylated CETP with reduced functionality, and larger HDL than healthy controls (122). CETP is a minor component of HDL, whose function is to temporarily associate with HDL while bridging between the HDL and ApoB particle between which the exchange of lipids occurs, thus it is often missed as an HDL-associated protein depending on the HDL isolation method and sensitivity of the protein detection method (24). However, its importance in lipid metabolism and strong links with CVD make it an important protein whose content and glycosylation when associated with HDL particles is an area of focus for future studies.

### Phospholipid Transfer Protein

The primary function of PLTP is to transfer phospholipids from ApoB containing TG-rich lipoproteins to HDL (123, 124). As a key modulator of HDL size, composition, and concentration PLTP has gained considerable attention for its role in the development of CVD (125). PLTP overexpression has been reported to be an independent risk factor for CAD and is associated with type II diabetes and obesity (126). Two forms of PLTP have been described that have high and low phospholipid transfer activity, which may explain the conflicting findings of the association between PLTP and pro- vs. anti-atherogenic effects (127). Higher concentrations of the low-activity PLTP type may be the driver of the pro-atherogenic effects, and PLTP glycosylation may play a critical role in the function and activity of the protein. Human PLTP has 6 N-linked and 2 O-linked glycoforms (123). Multiple earlier studies showed that tunicamycin treatment disrupts the ability of cells to secrete PTLP, suggesting glycosylation is necessary for synthesis and secretion (128, 129). A later study confirmed that inhibition of PLTP N-glycosylation affected its structural stability and markedly reduced its ability to be excreted resulting in the non-glycosylated PLTP being intracellularly degraded (125). Much like CETP, PLTP is a protein that temporarily associates with HDL particles to mediate the exchange of material between HDL and ApoB-containing lipoproteins, thus the ability to detect its presence on HDL depends on the nature of the HDL isolation method. Although PLTP is a minor constituent of HDL particles and thus measuring its glycosylation may be limited without enrichment prior to analysis, its content and glycosylation profile are likely to be important factors in overall HDL metabolism.

## Potential for Modifying The Glycosylation of HDL-Associated Proteins to Confer Therapeutic Value

Given the growing evidence that HDL glycosylation may be critically involved in both metabolism and function, with implications for both CVD diagnosis and treatment, the potential for HDL-based therapeutics targeting HDL glycosylation is compelling. Strategies to reduce CVD risk and prevent or reverse CVD by increasing the concentration of HDL particles have been largely disappointing. Increasing the number of HDL particles through pharmacological means (e.g., CETP inhibitors, niacin), has met with some success, however the ability to further reduce residual CVD risk following LDL-lowering with statins has been difficult to achieve (130, 131). Several additional HDL modifying therapies, including injection with reconstituted HDL particles, ApoA-I, as well as extracorporeal HDL lipid depletion, where HDL particles are removed from plasma, exogenously delipidated, and then reinfused, have similarly met with modest success despite promising results in animal trials (132, 133). Thus, novel therapeutic approaches to increase not just the concentration but also the function of HDL particles remain an important area of research. The potential for dietary and pharmacological strategies to improve HDL function via modulation of HDL glycoprofiles is tantalizing given the growing evidence of the importance of HDL glycosylation in its function. Several recent studies show promising results for the modification of HDL glycosylation through diet. Whereas, the glycosylation of HDL-associated ApoE was not affected by a short-term intervention with Mediterranean vs. fast food diet, the glycosylation of HDL-associated ApoC-III was significantly altered in just 4 days (87). Specifically, disialylated ApoC-III (ApoC-III_2_) was increased after the Mediterranean diet whereas nonsialylated ApoC-III (ApoC-III_0_) was increased after 4 days of consuming a diet enriched in saturated fat and simple sugars and depleted in fiber (87). These alterations were associated with HDL cholesterol efflux capacity as well as immunomodulatory capacity (ability to suppress cytokine secretion in stimulated monocytes) (87). In young children in Ghana supplemented with a lipid nutrient supplement, HDL glycopeptides that were altered by the supplement were correlated with HDL cholesterol efflux capacity (25). There is also evidence that targeting GALNT2 activity may be a viable strategy to alter the glycosylation of HDL-associated proteins and thus increase HDL concentration and function (64, 134). While this research area is very new, early tantalizing evidence provides support for the idea that the alteration of HDL glycoprofiles via dietary or pharmacological interventions may be a viable strategy for improving the functional capacity of HDL particles and thus improving CV outcomes.

## Conclusion

While the study of HDL glycosylation is still in a nascent state, emerging evidence suggests that differential glycoprofiles of HDL-associated proteins may be diagnostic and may reveal new mechanisms in lipoprotein-mediated aspects of CVD. In order to uncover glycan-based disease biomarkers newly developed glycan analytical methods need to be applied to large, comprehensively characterized, and preferably genotyped cohorts with known CV outcomes. Basic cell and molecular biology studies are also needed to better understand how glycosylation affects HDL metabolism and function, so that the potential for modifying the glycosylation of HDL-associated proteins through intervention to confer therapeutic value can be realized. In the last 10 years there has been progress toward developing the fundamental methodologies for both the isolation of HDL from plasma and the analysis of HDL glycosylation especially using MS. This field is now ripe for major discoveries utilizing these tools in the areas of glycan-based HDL CVD biomarkers, novel CVD disease mechanisms, and ultimately, novel HDL-based therapeutics for cardioprotection.

## Author Contributions

ER and AZ had significant contribution to the content, design, and preparation of this manuscript. Both authors have reviewed and approved the submission of this document.

## Funding

The project described was supported by the National Institute of Aging of the NIH (R01AG062240, UH3CA241694) and the USDA National Institute of Food and Agriculture, Hatch project (CA-D-NUT-2242-H). The content is solely the responsibiltiy of the authors and does not necessarily represent the official views of the NIH.

## Conflict of Interest

The authors declare that the research was conducted in the absence of any commercial or financial relationships that could be construed as a potential conflict of interest.

## Publisher's Note

All claims expressed in this article are solely those of the authors and do not necessarily represent those of their affiliated organizations, or those of the publisher, the editors and the reviewers. Any product that may be evaluated in this article, or claim that may be made by its manufacturer, is not guaranteed or endorsed by the publisher.

## References

[1] Kannel WB Castelli WP McNamara PM. Serum lipid fractions and risk of coronary heart disease. The Framingham study. Minn Med. (1969) 52:1225–30. 10.1378/chest.56.1.43 5804989

[2] Gordon DJ Probstfield JL Garrison RJ Neaton JD Castelli WP Knoke JD. High-density lipoprotein cholesterol and cardiovascular disease. Four prospective American studies. Circulation. (1989) 79:8–15. 10.1161/01.CIR.79.1.8 2642759

[3] Miller GJ Miller NE. Plasma-high-density-lipoprotein concentration and development of ischæmic heart-disease. The Lancet. (1975) 305:16–9. 10.1016/S0140-6736(75)92376-4 46338

[4] Kim DS Burt AA Rosenthal EA Ranchalis JE Eintracht JF Hatsukami TS. HDL-3 is a superior predictor of carotid artery disease in a case-control cohort of Participants. J Am Heart Assoc. (1725) 3:e000902. 10.1161/JAHA.114.000902 24965026 PMC4309059

[5] Zheng JJ Agus JK Hong BV Tang X Rhodes CH Houts HE. Isolation of HDL by sequential flotation ultracentrifugation followed by size exclusion chromatography reveals size-based enrichment of HDL-associated proteins. Sci Rep. (2021) 11:16086. 10.1038/s41598-021-95451-3 34373542 PMC8352908

[6] Huang J Lee H Zivkovic AM Smilowitz JT Rivera N German JB. Glycomic analysis of high density lipoprotein shows a highly sialylated particle. J Proteome Res. (2014) 13:681–91. 10.1021/pr4012393 24417605 PMC3975653

[7] Krishnan S Huang J Lee H Guerrero A Berglund L Anuurad E. Combined high-density lipoprotein proteomic and glycomic profiles in patients at risk for coronary artery disease. J Proteome Res. (2015) 14:5109–18. 10.1021/acs.jproteome.5b00730 26535788

[8] Savinova OV Fillaus K Jing L Harris WS Shearer GC. Reduced apolipoprotein glycosylation in patients with the metabolic syndrome. PLoS ONE. (2014) 9:e104833. 10.1371/journal.pone.0104833 25118169 PMC4130598

[9] Sanllorente A Castañer O Lassale C Almanza-Aguilera E Elosua R Vila J. High-density lipoprotein functional traits and coronary artery disease in a general population: a case–cohort study. Eur J Prev Cardiol. (2022) 29:e47–9. 10.1093/eurjpc/zwaa149 33624097

[10] Eren E Yilmaz N Aydin O. High density lipoprotein and it's dysfunction. Open Biochem J. (2012) 6:78–93. 10.2174/1874091X01206010078 22888373 PMC3414806

[11] Liu D Ji L Zhang D Tong X Pan B Liu P. Nonenzymatic glycation of high-density lipoprotein impairs its anti-inflammatory effects in innate immunity. Diabetes Metab Res Rev. (2012) 28:186–95. 10.1002/dmrr.1297 21928330

[12] Van Lenten BJ Wagner AC Nayak DP Hama S Navab M Fogelman AM. High-density lipoprotein loses its anti-inflammatory properties during acute influenza a infection. Circulation. (2001) 103:2283–8. 10.1161/01.CIR.103.18.2283 11342478

[13] Tölle M Huang T Schuchardt M Jankowski V Prüfer N Jankowski J. High-density lipoprotein loses its anti-inflammatory capacity by accumulation of pro-inflammatory-serum amyloid A. Cardiovasc Res. (2012) 94:154–62. 10.1093/cvr/cvs089 22328092

[14] Maverakis E Kim K Shimoda M Gershwin ME Patel F Wilken R. Glycans in the immune system and the altered glycan theory of autoimmunity: a critical review. J Autoimmun. (2015) 0:1–13. 10.1016/j.jaut.2014.12.002 25578468 PMC4340844

[15] Kailemia MJ Wei W Nguyen K Beals E Sawrey-Kubicek L Rhodes C. Targeted measurements of O- and N-glycopeptides show that proteins in high density lipoprotein particles are enriched with specific glycosylation compared to plasma. J Proteome Res. (2018) 17:834–45. 10.1021/acs.jproteome.7b00604 29212317 PMC6343480

[16] Liu YS Guo XY Hirata T Rong Y Motooka D Kitajima T. N-Glycan–dependent protein folding and endoplasmic reticulum retention regulate GPI-anchor processing. J Cell Biol. (2017) 217:585–99. 10.1083/jcb.201706135 29255114 PMC5800811

[17] Ermonval M Duvet S Zonneveld D Cacan R Buttin G Braakman I. Truncated N-glycans affect protein folding in the ER of CHO-derived mutant cell lines without preventing calnexin binding. Glycobiology. (2000) 10:77–87. 10.1093/glycob/10.1.77 10570226

[18] Mauger JF Couture P Bergeron N Lamarche B. Apolipoprotein C-III isoforms: kinetics and relative implication in lipid metabolism. J Lipid Res. (2006) 47:1212–8. 10.1194/jlr.M500455-JLR200 16495512

[19] Krautter F Iqbal AJ. Glycans and glycan-binding proteins as regulators and potential targets in leukocyte recruitment. Front Cell Dev Biol. (2021) 9:624082. 10.3389/fcell.2021.624082 33614653 PMC7890243

[20] Varki A. Biological roles of glycans. Glycobiology. (2017) 27:3–49. 10.1093/glycob/cww086 27558841 PMC5884436

[21] Allard-Ratick MP. Everything in Moderation: Investigating the U-Shaped Link Between HDL Cholesterol Adverse Outcomes. (2019). Available online at: https://www.uscjournal.com/articles/everything-moderation-investigating-u-shaped-link-between-hdl-cholesterol-and-adverse (accessed April 17, 2022).

[22] Lorkowski SW Smith JD. HDL is not dead yet. Biomedicines. (2022) 10:128. 10.3390/biomedicines10010128 35052806 PMC8773442

[23] Yang HS Jeong HJ Kim H Hwang HK Hur M Lee S. Sex-specific U-shaped relationships between high-density lipoprotein cholesterol levels and 10-year major adverse cardiovascular events: a nationwide cohort study of 5.7 million South Koreans. Ann Lab Med. (2022) 42:415–27. 10.3343/alm.2022.42.4.415 35177562 PMC8859558

[24] Davidson WS Shah AS Sexmith H Gordon SM. The HDL proteome watch: compilation of studies leads to new insights on HDL function. Biochim Biophys Acta Mol Cell Biol Lipids. (2022) 1867:159072. 10.1016/j.bbalip.2021.159072 34800735 PMC8715479

[25] Hong BV Zhu C Wong M Sacchi R Rhodes CH Kang JW. Lipid-based nutrient supplementation increases high-density lipoprotein (HDL) cholesterol efflux capacity and is associated with changes in the HDL glycoproteome in children. ACS Omega. (2021) 6:32022–31. 10.1021/acsomega.1c04811 34870025 PMC8638293

[26] Vaisar t. Proteomics investigations of HDL, challenges and promise. Curr Vasc Pharmacol. (2012) 10:410–21. 10.2174/157016112800812755 22339300 PMC3685576

[27] Heinecke JW. The HDL proteome: a marker–and perhaps mediator–of coronary artery disease. J Lipid Res. (2009) 50(Suppl):S167–71. 10.1194/jlr.R800097-JLR200 19060251 PMC2674735

[28] Vickers KC Palmisano BT Shoucri BM Shamburek RD Remaley AT. MicroRNAs are transported in plasma and delivered to recipient cells by high-density lipoproteins. Nat Cell Biol. (2011) 13:423–33. 10.1038/ncb2210 21423178 PMC3074610

[29] Michell DL Allen RM Landstreet SR Zhao S Toth CL Sheng Q. Isolation of High-density lipoproteins for Non-coding Small RNA quantification. J Vis Exp. (2016) 54488. 10.3791/54488 27929461 PMC5226318

[30] Holzer M Kern S Birner-Grünberger R Curcic S Heinemann A Marsche G. Refined purification strategy for reliable proteomic profiling of HDL2/3: impact on proteomic complexity. Sci Rep. (2016) 6:38533. 10.1038/srep38533 27917957 PMC5137140

[31] Hua S Nwosu CC Strum JS Seipert RR An HJ Zivkovic AM. Site-specific protein glycosylation analysis with glycan isomer differentiation. Anal Bioanal Chem. (2012) 403:1291–302. 10.1007/s00216-011-5109-x 21647803

[32] Ruhaak LR Xu G Li Q Goonatilleke E Lebrilla CB. Mass spectrometry approaches to glycomic and glycoproteomic analyses. Chem Rev. (2018) 118:7886–930. 10.1021/acs.chemrev.7b00732 29553244 PMC7757723

[33] Xie Y Chen S Li Q Sheng Y Alvarez MR Reyes J. Glycan–protein cross-linking mass spectrometry reveals sialic acid-mediated protein networks on cell surfaces. Chem Sci. (2021) 12:8767–77. 10.1039/D1SC00814E 34257876 PMC8246274

[34] Kim T Xie Y Li Q Artegoitia VM Lebrilla CB Keim NL. Diet affects glycosylation of serum proteins in women at risk for cardiometabolic disease. Eur J Nutr. (2021) 60:3727–41. 10.1007/s00394-021-02539-7 33770218 PMC8437848

[35] Banazadeh A Veillon L Wooding KM Zabet M Mechref Y. Recent advances in mass spectrometric analysis of glycoproteins. Electrophoresis. (2017) 38:162–89. 10.1002/elps.201600357 27757981 PMC5221507

[36] Nwosu CC Seipert RR Strum JS Hua SS An HJ Zivkovic AM. Simultaneous and extensive site-specific N- and O-glycosylation analysis in protein mixtures. J Proteome Res. (2011) 10:2612–24. 10.1021/pr2001429 21469647 PMC3097320

[37] Krishnan S Shimoda M Sacchi R Kailemia MJ Luxardi G Kaysen GA. HDL glycoprotein composition and site-specific glycosylation differentiates between clinical groups and affects IL-6 secretion in lipopolysaccharide-stimulated monocytes. Sci Rep. (2017) 7:43728. 10.1038/srep43728 28287093 PMC5347119

[38] Buszewski B Noga S. Hydrophilic interaction liquid chromatography (HILIC)—a powerful separation technique. Anal Bioanal Chem. (2012) 402:231–47. 10.1007/s00216-011-5308-5 21879300 PMC3249561

[39] Zvintzou E Lhomme M Chasapi S Filou S Theodoropoulos V Xapapadaki E. Pleiotropic effects of apolipoprotein C3 on HDL functionality and adipose tissue metabolic activity. J Lipid Res. (2017) 58:1869–83. 10.1194/jlr.M077925 28701354 PMC5580900

[40] Gudelj I Lauc G. Protein N-glycosylation in cardiovascular diseases and related risk factors. Curr Cardiovasc Risk Rep. (2018) 12:16. 10.1007/s12170-018-0579-429470411

[41] Okoro EU Zhao Y Guo Z Zhou L Lin X Yang H. Apolipoprotein E4 is deficient in inducing macrophage ABCA1 expression and stimulating the Sp1 signaling pathway. PLoS ONE. (2012) 7:e44430. 10.1371/journal.pone.0044430 22984509 PMC3439389

[42] Furtado JD Yamamoto R Melchior JT Andraski AB Gamez-Guerrero M Mulcahy P. Distinct proteomic signatures in 16 HDL (high-density lipoprotein) subspecies. Arterioscler Thromb Vasc Biol. (2018) 38:2827–42. 10.1161/ATVBAHA.118.311607 30571168 PMC6309805

[43] Cubedo J Padró T Badimon L. Glycoproteome of human apolipoprotein A-I: N- and O-glycosylated forms are increased in patients with acute myocardial infarction. Transl Res. (2014) 164:209–22. 10.1016/j.trsl.2014.03.008 24709669

[44] Lapolla A Brioschi M Banfi C Tremoli E Cosma C Bonfante L. Nonenzymatically glycated lipoprotein ApoA-I in plasma of diabetic and nephropathic patients. Ann N Y Acad Sci. (2008) 1126:295–9. 10.1196/annals.1433.005 18079481

[45] Pirillo A Svecla M Catapano AL Holleboom AG Norata GD. Impact of protein glycosylation on lipoprotein metabolism and atherosclerosis. Cardiovasc Res. (2021) 117:1033–45. 10.1093/cvr/cvaa252 32886765

[46] Syvänne M Kahri J Virtanen KS Taskinen MR. HDLs containing apolipoproteins A-I and A-II (LpA-I:A-II) as markers of coronary artery disease in men with non-insulin-dependent diabetes mellitus. Circulation. (1995) 92:364–70. 10.1161/01.CIR.92.3.364 7634450

[47] Alaupovic P Mack WJ Knight-Gibson C Hodis HN. The role of triglyceride-rich lipoprotein families in the progression of atherosclerotic lesions as determined by sequential coronary angiography from a controlled clinical trial. Arterioscler Thromb Vasc Biol. (1997) 17:715–22. 10.1161/01.ATV.17.4.715 9108785

[48] Birjmohun RS Dallinga-Thie GM Kuivenhoven JA Stroes ESG Otvos JD Wareham NJ. Apolipoprotein A-II is inversely associated with risk of future coronary artery disease. Circulation. (2007) 116:2029–35. 10.1161/CIRCULATIONAHA.107.704031 17923573

[49] Pussinen PJ Jauhiainen M Metso J Pyle LE Marcel YL Fidge NH. Binding of phospholipid transfer protein (PLTP) to apolipoproteins A-I and A-II: location of a PLTP binding domain in the amino terminal region of apoA-I. J Lipid Res. (1998) 39:152–61. 10.1016/S0022-2275(20)34211-5 9469594

[50] Gao X Yuan S Jayaraman S Gursky O. Role of apolipoprotein A-II in the structure and remodeling of human high-density lipoprotein (HDL): protein conformational ensemble on HDL. Biochemistry. (2012) 51:4633–41. 10.1021/bi300555d 22631438 PMC5603225

[51] Melchior JT Street SE Andraski AB Furtado JD Sacks FM Shute RL. Apolipoprotein A-II alters the proteome of human lipoproteins and enhances cholesterol efflux from ABCA1. J Lipid Res. (2017) 58:1374–85. 10.1194/jlr.M075382 28476857 PMC5496035

[52] Jin Y Manabe T. Direct targeting of human plasma for matrix-assisted laser desorption/ionization and analysis of plasma proteins by time of flight-mass spectrometry. Electrophoresis. (2005) 26:2823–34. 10.1002/elps.200410421 15934056

[53] Remaley AT Wong AW Schumacher UK Meng MS Brewer HB Hoeg JM. O-linked glycosylation modifies the association of apolipoprotein A-II to high density lipoproteins. J Biol Chem. (1993) 268:6785–90. 10.1016/S0021-9258(18)53318-4 8454651

[54] Norata GD Tsimikas S Pirillo A Catapano AL. Apolipoprotein C-III: from pathophysiology to pharmacology. Trends Pharmacol Sci. (2015) 36:675–87. 10.1016/j.tips.2015.07.001 26435212

[55] Taskinen MR Borén J. Why is apolipoprotein CIII emerging as a novel therapeutic target to reduce the burden of cardiovascular disease? Curr Atheroscler Rep. (2016) 18:59. 10.1007/s11883-016-0614-1 27613744 PMC5018018

[56] Dittrich J Beutner F Teren A Thiery J Burkhardt R Scholz M. Plasma levels of apolipoproteins C-III, A-IV, and E are independently associated with stable atherosclerotic cardiovascular disease. Atherosclerosis. (2019) 281:17–24. 10.1016/j.atherosclerosis.2018.11.006 30594773

[57] Vaisar T Mayer P Nilsson E Zhao XQ Knopp R Prazen BJ. HDL in humans with cardiovascular disease exhibits a proteomic signature. Clin Chim Acta. (2010) 411:972–9. 10.1016/j.cca.2010.03.023 20307520 PMC2862883

[58] Jensen MK Rimm EB Furtado JD Sacks FM. Apolipoprotein C-III as a potential modulator of the association between HDL-cholesterol and incident coronary heart disease. J Am Heart Assoc. (2012) 1:jah3-e000232. 10.1161/JAHA.111.000232 23130121 PMC3487368

[59] Kornak U Reynders E Dimopoulou A van Reeuwijk J Fischer B Rajab A. Impaired glycosylation and cutis laxa caused by mutations in the vesicular H+-ATPase subunit ATP6V0A2. Nat Genet. (2008) 40:32–4. 10.1038/ng.2007.45 18157129

[60] Juntti-Berggren L Refai E Appelskog I Andersson M Imreh G Dekki N. Apolipoprotein CIII promotes Ca2+-dependent beta cell death in type 1 diabetes. Proc Natl Acad Sci U S A. (2004) 101:10090–4. 10.1073/pnas.0403551101 15210953 PMC454169

[61] Harvey SB Zhang Y Wilson-Grady J Monkkonen T Nelsestuen GL Kasthuri RS. O-Glycoside biomarker of apolipoprotein C3: responsiveness to obesity, bariatric surgery, and therapy with metformin, to chronic or severe liver disease and to mortality in severe sepsis and graft vs. host disease. J Proteome Res. (2009) 8:603–12. 10.1021/pr800751x 19055479

[62] Ueda K Fukase Y Katagiri T Ishikawa N Irie S Sato TA. Targeted serum glycoproteomics for the discovery of lung cancer-associated glycosylation disorders using lectin-coupled ProteinChip arrays. Proteomics. (2009) 9:2182–92. 10.1002/pmic.200800374 19322776

[63] Zilmer M Edmondson AC Khetarpal SA Alesi V Zaki MS Rostasy K. Novel congenital disorder of O-linked glycosylation caused by GALNT2 loss of function. Brain. (2020) 143:1114–26. 10.1093/brain/awaa063 32293671 PMC7534148

[64] Khetarpal SA Schjoldager KT Christoffersen C Raghavan A Edmondson AC Reutter HM. Loss of function of GALNT2 lowers high density lipoproteins in humans, nonhuman primates, and rodents. Cell Metab. (2016) 24:234–45. 10.1016/j.cmet.2016.07.012 27508872 PMC5663192

[65] Dallinga-Thie GM Kroon J Borén J Chapman MJ. Triglyceride-rich lipoproteins and remnants: targets for therapy? Curr Cardiol Rep. (2016) 18:67. 10.1007/s11886-016-0745-6 27216847 PMC4877422

[66] van Capelleveen JC Moens SJB Yang X Kastelein JJP Wareham NJ Zwinderman AH. Apolipoprotein C-III levels and incident coronary artery disease risk: the EPIC-norfolk prospective population study. Arterioscler Thromb Vasc Biol. (2017) 37:1206–12. 10.1161/ATVBAHA.117.309007 28473441 PMC5484077

[67] Jørgensen AB Frikke-Schmidt R Nordestgaard BG Tybjærg-Hansen A. Loss-of-function mutations in APOC3 and risk of ischemic vascular disease. N Engl J Med. (2014) 371:32–41. 10.1056/NEJMoa1308027 24941082

[68] Koska J Yassine H Trenchevska O Sinari S Schwenke DC Yen FT. Disialylated apolipoprotein C-III proteoform is associated with improved lipids in prediabetes and type 2 diabetes1. J Lipid Res. (2016) 57:894–905. 10.1194/jlr.P064816 26945091 PMC4847634

[69] Kegulian NC Ramms B Horton S Trenchevska O Nedelkov D Graham MJ. ApoC-III glycoforms are differentially cleared by hepatic triglyceride-rich lipoprotein receptors. Arterioscler Thromb Vasc Biol. (2019) 39:2145–56. 10.1161/ATVBAHA.119.312723 31390883 PMC6761044

[70] Roghani A Zannis VI. Mutagenesis of the glycosylation site of human ApoCIII. O-linked glycosylation is not required for ApoCIII secretion and lipid binding. J Biol Chem. (1988) 263:17925–32. 10.1016/S0021-9258(19)81305-4 3192519

[71] Egert S Rimbach G Huebbe P. ApoE genotype: from geographic distribution to function and responsiveness to dietary factors. Proc Nutr Soc. (2012) 71:410–24. 10.1017/S0029665112000249 22564824

[72] Corder EH Saunders AM Strittmatter WJ Schmechel DE Gaskell PC Small GW. Gene dose of apolipoprotein E type 4 allele and the risk of Alzheimer's disease in late onset families. Science. (1993) 261:921–3. 10.1126/science.8346443 8346443

[73] Marais AD. Apolipoprotein E in lipoprotein metabolism, health and cardiovascular disease. Pathology. (2019) 51:165–76. 10.1016/j.pathol.2018.11.002 30598326

[74] Mahoney-Sanchez L Belaidi AA Bush AI Ayton S. The complex role of apolipoprotein E in Alzheimer's disease: an overview and update. J Mol Neurosci. (2016) 60:325–35. 10.1007/s12031-016-0839-z 27647307

[75] Deane R Sagare A Hamm K Parisi M Lane S Finn MB. apoE isoform–specific disruption of amyloid β peptide clearance from mouse brain. J Clin Invest. (2008) 118:4002–13. 10.1172/JCI36663 19033669 PMC2582453

[76] Heeren J Grewal T Laatsch A Becker N Rinninger F Rye KA. Impaired recycling of apolipoprotein E4 is associated with intracellular cholesterol accumulation. J Biol Chem. (2004) 279:55483–92. 10.1074/jbc.M409324200 15485881

[77] Minagawa H Gong JS Jung CG Watanabe A Lund-Katz S Phillips MC. Mechanism underlying apolipoprotein E (ApoE) isoform-dependent lipid efflux from neural cells in culture. J Neurosci Res. (2009) 87:2498–508. 10.1002/jnr.22073 19326444 PMC3065888

[78] Mamotte CDS Sturm M Foo JI van Bockxmeer FM Taylor RR. Comparison of the LDL-receptor binding of VLDL and LDL from apoE4 and apoE3 homozygotes. Am J Physiol Endocrin Metab. (1999) 276:E553–7. 10.1152/ajpendo.1999.276.3.E553 10070023

[79] Röhrl C Stangl H. HDL endocytosis and resecretion. Biochim Biophys Acta. (2013) 1831:1626–33. 10.1016/j.bbalip.2013.07.014 23939397 PMC3795453

[80] Heeren J Beisiegel U Grewal T. Apolipoprotein E recycling. Arterioscler Thromb Vasc Biol. (2006) 26:442–8. 10.1161/01.ATV.0000201282.64751.47 16373604

[81] Marmillot P Rao MN Liu QH Lakshman MR. Desialylation of human apolipoprotein E decreases its binding to human high-density lipoprotein and its ability to deliver esterified cholesterol to the liver. Metabolism. (1999) 48:1184–92. 10.1016/S0026-0495(99)90136-1 10484062

[82] Jain RS Quarfordt SH. The carbohydrate content of apolipoprotein E from human very low density lipoproteins. Life Sci. (1979) 25:1315–23. 10.1016/0024-3205(79)90397-7 92742

[83] Zannis VI. vanderSpek J, Silverman D. Intracellular modifications of human apolipoprotein E. J Biol Chem. (1986) 261:13415–21. 10.1016/S0021-9258(18)67033-4 3020031

[84] Wernette-Hammond ME Lauer SJ Corsini A Walker D Taylor JM Rall SC. Glycosylation of human apolipoprotein E: the carbohydrate attachment site is threonine 194. J Biol Chem. (1989) 264:9094–101. 10.1016/S0021-9258(18)81907-X 2498325

[85] Mancone C Amicone L Fimia GM Bravo E Piacentini M Tripodi M. Proteomic analysis of human very low-density lipoprotein by two-dimensional gel electrophoresis and MALDI-TOF/TOF. Proteomics. (2007) 7:143–54. 10.1002/pmic.200600339 17154273

[86] Lee Y Kockx M Raftery MJ Jessup W Griffith R Kritharides L. Glycosylation and sialylation of macrophage-derived human apolipoprotein E analyzed by SDS-PAGE and mass spectrometry. Mol Cell Proteomics. (2010) 9:1968–81. 10.1074/mcp.M900430-MCP200 20511397 PMC2938105

[87] Zhu C Wong M Li Q Sawrey-Kubicek L Beals E Rhodes CH. Site-specific glycoprofiles of HDL-associated ApoE are correlated with HDL functional capacity and unaffected by short-term diet. J Proteome Res. (2019) 18:3977–84. 10.1021/acs.jproteome.9b00450 31545048 PMC7480961

[88] Fazio S Horie Y Weisgraber K Havekes L Rall S. Preferential association of apolipoprotein E Leiden with very low density lipoproteins of human plasma. J Lipid Res. (1993) 34:447–53. 10.1016/S0022-2275(20)40736-9 8468528

[89] Ghosh P Hale EA Mayur K Seddon J Lakshman MR. Effects of chronic alcohol treatment on the synthesis, sialylation, and disposition of nascent apolipoprotein E by peritoneal macrophages of rats. Am J Clin Nutr. (2000) 2:190–8. 10.1093/ajcn/72.1.190 10871579

[90] Flowers SA Grant OC Woods RJ Rebeck GW. O-glycosylation on cerebrospinal fluid and plasma apolipoprotein E differs in the lipid-binding domain. Glycobiology. (2020) 30:74–85. 10.1093/glycob/cwz084 31616924 PMC7335482

[91] Sugano M Yamauchi K Kawasaki K Tozuka M Fujita K Okumura N. Sialic acid moiety of apolipoprotein E3 at Thr194 affects its interaction with β-amyloid1–42 peptides. Clinica Chimica Acta. (2008) 388:123–9. 10.1016/j.cca.2007.10.024 18023277

[92] McCarthy C Saldova R Wormald MR Rudd PM McElvaney NG Reeves EP. The role and importance of glycosylation of acute phase proteins with focus on alpha-1 antitrypsin in acute and chronic inflammatory conditions. J Proteome Res. (2014) 13:3131–43. 10.1021/pr500146y 24892502

[93] Gordon SM McKenzie B Kemeh G Sampson M Perl S Young NS. Rosuvastatin alters the proteome of high density lipoproteins: generation of alpha-1-antitrypsin enriched particles with anti-inflammatory properties. Mol Cell Proteomics. (2015) 14:3247–57. 10.1074/mcp.M115.054031 26483418 PMC4762624

[94] Lechowicz U Rudzinski S Jezela-Stanek A Janciauskiene S Chorostowska-Wynimko J. Post-translational modifications of circulating alpha-1-antitrypsin protein. Int J Mol Sci. (2020) 21:9187. 10.3390/ijms21239187 33276468 PMC7731214

[95] Liang Y Ma T Thakur A Yu H Gao L Shi P. Differentially expressed glycosylated patterns of α-1-antitrypsin as serum biomarkers for the diagnosis of lung cancer. Glycobiology. (2015) 25:331–40. 10.1093/glycob/cwu115 25347993

[96] McElvaney OJ McEvoy NL McElvaney OF Carroll TP Murphy MP Dunlea DM. Characterization of the inflammatory response to severe COVID-19 illness. Am J Respir Crit Care Med. (2020) 202:812–21. 10.1164/rccm.202005-1583OC 32584597 PMC7491404

[97] Gross V vom Berg D Kreuzkamp J Ganter U Bauer J Würtemberger G. Biosynthesis and secretion of M- and Z-type alpha 1-proteinase inhibitor by human monocytes Effect of inhibitors of glycosylation and of oligosaccharide processing on secretion and function. Biol Chem Hoppe Seyler. (1990) 371:231–8. 10.1515/bchm3.1990.371.1.231 2111144

[98] Jeppsson JO Larsson C Eriksson S. Characterization of α1-antitrypsin in the inclusion bodies from the liver in α1-antitrypsin deficiency. N Engl J Med. (1975) 293:576–9. 10.1056/NEJM197509182931203 168490

[99] Saitoh A Aoyagi Y Asakura H. Structural analysis on the sugar chains of human α1-antitrypsin: presence of fucosylated biantennary glycan in hepatocellular carcinoma. Arch Biochem Biophys. (1993) 303:281–7. 10.1006/abbi.1993.1284 8390218

[100] Ketteler M Bongartz P Westenfeld R Wildberger JE Mahnken AH Böhm R. Association of low fetuin-A (AHSG) concentrations in serum with cardiovascular mortality in patients on dialysis: a cross-sectional study. Lancet. (2003) 361:827–33. 10.1016/S0140-6736(03)12710-9 12642050

[101] Ketteler M Schlieper G Floege J. Calcification and cardiovascular health. Hypertension. (2006) 47:1027–34. 10.1161/01.HYP.0000219635.51844.da 16618842

[102] Jahnen-Dechent W Heiss A Schäfer C Ketteler M Towler DA. Fetuin-A Regulation of calcified matrix metabolism. Circ Res. (2011) 108:1494–509. 10.1161/CIRCRESAHA.110.234260 21659653

[103] Ix JH Barrett-Connor E Wassel CL Cummins K Bergstrom J Daniels LB. The associations of fetuin-A with subclinical cardiovascular disease in community-dwelling persons: the rancho bernardo study. J Am Coll Cardiol. (2011) 58:2372–9. 10.1016/j.jacc.2011.08.035 22115642 PMC3224791

[104] Sarrats A Saldova R Pla E Fort E Harvey DJ Struwe WB. Glycosylation of liver acute-phase proteins in pancreatic cancer and chronic pancreatitis. Proteomics Clin Appl. (2010) 4:432–48. 10.1002/prca.200900150 21137062

[105] Sorci-Thomas MG Bhat S Thomas MJ. Activation of lecithin:cholesterol acyltransferase by HDL ApoA-I central helices. Clin Lipidol. (2009) 4:113–24. 10.2217/17584299.4.1.113 20582235 PMC2891274

[106] Yokoyama K Tani S Matsuo R Matsumoto N. Association of lecithin-cholesterol acyltransferase activity and low-density lipoprotein heterogeneity with atherosclerotic cardiovascular disease risk: a longitudinal pilot study. BMC Cardiovasc Disord. (2018) 18:224. 10.1186/s12872-018-0967-1 30518338 PMC6280370

[107] Manthei KA Patra D Wilson CJ Fawaz MV Piersimoni L Shenkar JC. Structural analysis of lecithin:cholesterol acyltransferase bound to high density lipoprotein particles. Commun Biol. (2020) 3:1–11. 10.1038/s42003-019-0749-z 31942029 PMC6962161

[108] Hovingh GK Hutten BA Holleboom AG Petersen W Rol P Stalenhoef A. Compromised LCAT function is associated with increased atherosclerosis. Circulation. (2005) 112:879–84. 10.1161/CIRCULATIONAHA.105.540427 16061733

[109] Lacko AG Reason AJ Nuckolls C Kudchodkar BJ Nair MP Sundarrajan G. Characterization of recombinant human plasma lecithin: cholesterol acyltransferase (LCAT): N-linked carbohydrate structures and catalytic properties. J Lipid Res. (1998) 39:807–20. 10.1016/S0022-2275(20)32568-2 9555945

[110] Schindler PA Settineri CA Collet X Fielding CJ Burlingame AL. Site-specific detection and structural characterization of the glycosylation of human plasma proteins lecithin: cholesterol acyltransferase and apolipoprotein D using HPLC/electrospray mass spectrometry and sequential glycosidase digestion. Protein Sci. (1995) 4:791–803. 10.1002/pro.5560040419 7613477 PMC2143102

[111] Miller KR Wang J Sorci-Thomas M Anderson RA Parks JS. Glycosylation structure and enzyme activity of lecithin: cholesterol acyltransferase from human plasma, HepG2 cells, and baculoviral and Chinese hamster ovary cell expression systems. J Lipid Res. (1996) 37:551–61. 10.1016/S0022-2275(20)37598-2 8728318

[112] Kosman J Jonas A. Deletion of specific glycan chains affects differentially the stability, local structures, and activity of lecithin-cholesterol acyltransferase^*^. J Biol Chem. (2001) 276:37230–6. 10.1074/jbc.M104326200 11486003

[113] O K Hill JS Wang X McLeod R Pritchard PH. Lecithin: cholesterol acyltransferase: role of N-linked glycosylation in enzyme function. Biochem J. (1993) 294:879–84. 10.1042/bj2940879 8379944 PMC1134544

[114] Sukhorukov V Gudelj I Pučić-Baković M Zakiev E Orekhov A Kontush A. Glycosylation of human plasma lipoproteins reveals a high level of diversity, which directly impacts their functional properties. Biochimica et Biophysica Acta (BBA) – Mol Cell Biol Lipids. (2019) 1864:643–53. 10.1016/j.bbalip.2019.01.005 30641224

[115] Carlson LA Holmquist L. Evidence for deficiency of high density lipoprotein lecithin: cholesterol acyltransferase activity (alpha-LCAT) in fish eye disease. Acta Med Scand. (1985) 218:189–96. 10.1111/j.0954-6820.1985.tb08846.x 4061122

[116] Barter PJ Brewer HB Chapman MJ Hennekens CH Rader DJ Tall AR. Cholesteryl ester transfer protein. Arterioscler Thromb Vasc Biol. (2003) 23:160–7. 10.1161/01.ATV.0000054658.91146.64 12588754

[117] Ikewaki K Rader DJ Sakamoto T Nishiwaki M Wakimoto N Schaefer JR. Delayed catabolism of high density lipoprotein apolipoproteins A-I and A-II in human cholesteryl ester transfer protein deficiency. J Clin Invest. (1993) 92:1650–8. 10.1172/JCI116750 8408618 PMC288323

[118] Holmes MV Davey Smith G. Revealing the effect of CETP inhibition in cardiovascular disease. Nat Rev Cardiol. (2017) 14:635–6. 10.1038/nrcardio.2017.156 28980665 PMC5644574

[119] Stevenson SC Wang S Deng L Tall AR. Human plasma cholesteryl ester transfer protein consists of a mixture of two forms reflecting variable glycosylation at asparagine 341. Biochemistry. (1993) 32:5121–6. 10.1021/bi00070a021 8494888

[120] Tall A. Plasma cholesteryl ester transfer protein. J Lipid Res. (1993) 34:1255–74. 10.1016/S0022-2275(20)36957-1 8409761

[121] Liinamaa MJ Hannuksela ML Rämet ME Savolainen MJ. Defective glycosylation of cholesteryl ester transfer protein in plasma from alcohol abusers. Alcohol Alcohol. (2006) 41:18–23. 10.1093/alcalc/agh216 16203750

[122] van den Boogert MAW Crunelle CL Ali L Larsen LE Kuil SD Levels JHM. Reduced CETP glycosylation and activity in patients with homozygous B4GALT1 mutations. J Inherit Metab Dis. (2020) 43:611–7. 10.1002/jimd.12200 31800099 PMC7318693

[123] Day JR Albers JJ Lofton-Day CE Gilbert TL Ching AF Grant FJ. Complete cDNA encoding human phospholipid transfer protein from human endothelial cells. J Biol Chem. (1994) 269:9388–91. 10.1016/S0021-9258(17)37120-X 8132678

[124] Jiang XC Bruce C. Regulation of murine plasma phospholipid transfer protein activity and mRNA levels by lipopolysaccharide and high cholesterol diet (*). J Biol Chem. (1995) 270:17133–8. 10.1074/jbc.270.29.17133 7615508

[125] Huuskonen J Olkkonen VM Jauhiainen M Ehnholm C. The impact of phospholipid transfer protein (PLTP) on HDL metabolism. Atherosclerosis. (2001) 155:269–81. 10.1016/S0021-9150(01)00447-6 11254896

[126] Schlitt A Bickel C Thumma P Blankenberg S Rupprecht HJ Meyer J. High plasma phospholipid transfer protein levels as a risk factor for coronary artery disease. Arterioscler Thromb Vasc Biol. (2003) 23:1857–62. 10.1161/01.ATV.0000094433.98445.7F 12947020

[127] Krauss RM. Phospholipid transfer protein and atherosclerosis. Circulation. (2010) 122:452–4. 10.1161/CIRCULATIONAHA.110.966572 20644011

[128] Au-Young J Fielding CJ. Synthesis and secretion of wild-type and mutant human plasma cholesteryl ester transfer protein in baculovirus-transfected insect cells: the carboxyl-terminal region is required for both lipoprotein binding and catalysis of transfer. Proc Natl Acad Sci USA. (1992) 89:4094–8. 10.1073/pnas.89.9.4094 1570336 PMC525639

[129] Swenson T Simmons J Hesler C Bisgaier C Tall A. Cholesteryl ester transfer protein is secreted by Hep G2 cells and contains asparagine-linked carbohydrate and sialic acid. J Biol Chem. (1987) 262:16271–4. 10.1016/S0021-9258(18)49249-6 3316217

[130] D'Andrea E Hey SP Ramirez CL Kesselheim AS. Assessment of the role of niacin in managing cardiovascular disease outcomes: a systematic review and meta-analysis. JAMA Netw Open. (2019) 2:e192224. 10.1001/jamanetworkopen.2019.2224 30977858 PMC6481429

[131] Taheri H Filion KB Windle SB Reynier P Eisenberg MJ. Cholesteryl ester transfer protein inhibitors and cardiovascular outcomes: a systematic review and meta-analysis of randomized controlled trials. CRD. (2020) 145:236–50. 10.1159/000505365 32172237

[132] Abudukeremu A Huang C Li H Sun R Liu X Wu X. Efficacy and safety of high-density lipoprotein/apolipoprotein a1 replacement therapy in humans and mice with atherosclerosis: a systematic review and meta-analysis. Front Cardiovasc Med. (2021) 8:700233. 10.3389/fcvm.2021.700233 34422927 PMC8377725

[133] Barter PJ Rye KA. Targeting high-density lipoproteins to reduce cardiovascular risk: what is the evidence? Clin Ther. (2015) 37:2716–31. 10.1016/j.clinthera.2015.07.021 26548324

[134] Di Paola R Marucci A Trischitta V. GALNT2 effect on HDL-cholesterol and triglycerides levels in humans: evidence of pleiotropy? Nutr Metab Cardiovasc Dis. (2017) 27:281–2. 10.1016/j.numecd.2016.11.006 28153384

